# Ki-67 With MRI in Predicting the Complete Pathological Response Post-neoadjuvant Chemotherapy

**DOI:** 10.7759/cureus.73469

**Published:** 2024-11-11

**Authors:** Mahesh Kolli, Agnes George, Sridevi Aoutla, Santosh Kishor Chandrasekar, Shyam Nikethen Girivasan, Ravi Teja Kolli

**Affiliations:** 1 Family Medicine, Apollo Hospitals, Chennai, IND; 2 Medicine, Apollo Medicals Private Limited, Chennai, IND; 3 Neurology, Baby Memorial Hospital, Kozhikode, IND; 4 Radiology, Shri Adithya Multi Speciality Hospital, Madurai, IND; 5 Medicine, University Hospital Ayr, Ayr, GBR; 6 Pharmacy, JSS College of Pharmacy, Ooty, IND; 7 Medical, Apollo Proton Cancer Centre, Chennai, IND

**Keywords:** ki-67 expression, mri breast, pathological complete response (pcr), triple-negative breast carcinoma, tumor size and shape

## Abstract

Neoadjuvant chemotherapy (NAC) is increasingly used for high-risk breast cancer to achieve pathologic complete response (pCR), an indicator of event-free survival and favorable survival outcomes. Integrating MRI and Ki-67 biomarker analysis into predictive models offers a promising approach to optimize NAC response assessment and guide personalized treatment strategies. This study evaluates the validity of combined MRI and Ki-67 metrics for predicting pCR. We conducted a systematic review and meta-analysis following Preferred Reporting Items for Systematic Reviews and Meta-Analysis (PRISMA) guidelines, including studies on NAC-treated breast cancer patients assessed by MRI and Ki-67. The predictive models were evaluated based on key parameters, including MRI-based tumor size reduction and Ki-67 levels, with outcomes measured by area under the receiver operating characteristic curve (AUC) and calibration metrics. Findings across ten studies consistently show that high Ki-67 levels and significant tumor size reduction on MRI are predictive of pCR, achieving AUCs near 0.90. The analysis highlighted that models integrating MRI with Ki-67 metrics outperformed single-modality approaches, showing enhanced predictive accuracy and calibration. However, high heterogeneity (I² = 77%) was noted, suggesting variability in imaging and Ki-67 assessment protocols across studies. This study underscores the combined utility of MRI and Ki-67 for the non-invasive prediction of pCR, offering both structural and biological insights into tumor responsiveness. The results align with prior research, affirming the role of Radiomic-clinicopathological models in providing a more comprehensive assessment compared to individual markers.

Further refinement of imaging and biomarker protocols could improve model reproducibility and applicability. Our findings highlight the robust predictive accuracy of MRI-Ki-67 integrated models for assessing pCR, marking a significant step toward personalized cancer care. Future studies should focus on refining these models with additional biomarkers and standardized protocols, facilitating their integration into routine clinical oncology to enhance treatment decision-making and patient outcomes.

## Introduction and background

Neoadjuvant chemotherapy (NAC) is increasingly used as first-line therapy for high-risk localized breast cancer, with studies showing no survival difference between neoadjuvant and adjuvant settings [1,2]. The conventional drug development process for cancers of the breast is being reevaluated, as neoadjuvant therapy offers an agile and effective strategy that utilizes pathological complete response (pCR) as a primary endpoint, allowing for the assessment of pharmacodynamic responses and predictive biomarkers [3]. FDA is now considering neoadjuvant trials for accelerated drug approval, particularly for high-risk tumors, while emphasizing the need for survival improvement during regular approval [3].

pCR is defined as the lack of invasive or in situ cancer in the resected breast tissue and regional lymph nodes following neoadjuvant therapy, based on hematoxylin and eosin staining [4,5]. Pathological response to preoperative therapy, though complex, has shown promising correlations with event-free and overall survival [6].

Ki-67 is a nuclear protein associated with cellular proliferation. While it is commonly used as a biomarker to evaluate tumor growth, the Ki-67 index is also used because it reflects the percentage of tumor cells actively dividing, offering insight into a tumor's aggressiveness [7]. Numerous studies indicate that a high Ki-67 index correlates with higher pCR rates after NAC in breast cancer, particularly in patients with aggressive tumor subtypes like human epidermal growth factor receptor 2 positive (HER2+) and triple-negative breast cancer (TNBC) [8-10].

MRI is also another valuable tool for assessing pCR after NAC, especially in TNBC, where it predicts response more accurately than in HR+HER2- subtypes [11]. Radiologic complete response (rCR) is defined as no visible contrast enhancement on T1-weighted MRI images; any residual enhancement indicates non-rCR (partial response, stable disease, or progression) per Response Evaluation Criteria in Solid Tumors (RECIST) 1.1. Residual lesion size is measured for correlation with pathology findings, aiding surgical and treatment planning [12].

Several studies show a positive correlation between pathological findings and MRI in predicting pCR in individuals treated with NAC for breast cancer [13-22], out of which few predictive radiomic-clinicopathological models had been developed for pCR prediction [13,14,18,21,22]. This study identifies and analyses the pooled data on available human studies performing MRI and pathological examination on breast cancer populations targeted to pCR on NAC utilization. The study aims to identify whether the Ki-67 index and MRI findings serve as sufficient radiomic-clinicopathological markers to predict and identify pCR.

## Review

Methodology

Selection Criteria

We conducted this study and meta-analysis following the Preferred Reporting Items for Systematic Reviews and Meta-Analysis (PRISMA) guidelines [23] and the Cochrane Handbook [24]. The inclusion and exclusion criteria are as follows.

Inclusion criteria: Participants (P) diagnosed with high-risk breast cancer, especially TNBC and HER2+ tumors, who received NAC. Interventions (I) included using MRI and the Ki-67 index as biomarkers to assess pCR following NAC. Comparators (C) included the outcomes in patients with pCR and non-pCR. Outcomes (O) included Ki-67 index, Ki-67 positive count in pCR and non-pCR groups, size of tumor post-NAC assessed by MRI between pCR and non-pCR groups, and radiomic-clinicopathological models predicting pCR. Study design (S) of the included studies were retrospective or prospective cohort studies with or without a randomization process, which were published from December 2010 to October 2024.

Exclusion criteria: Studies involving non-breast cancer diagnoses, those lacking TNBC or HER2+ subtypes when relevant for pCR prediction, or those not employing MRI or Ki-67 index for assessment were ruled. Studies without comparisons of the Ki-67 Index or MRI findings between pCR and non-pCR groups were also excluded, as were those not reporting pCR as a primary outcome when linked to prediction models. Case reports, reviews, non-human studies, and those not meeting PRISMA or Cochrane standards were excluded, along with studies with incomplete MRI or Ki-67 data related to pCR.

The inclusion and exclusion criteria were structured to ensure relevance and rigor in evaluating predictors of pCR in high-risk breast cancer, particularly in TNBC and HER2+ subtypes. By focusing on studies using MRI and Ki-67 as biomarkers, the criteria align with the aim of the study to assess minimal invasive and histopathological predictors of NAC response. Excluding studies on non-breast cancers, irrelevant subtypes, or those lacking comparative pCR analysis ensures that the selected studies directly contribute to understanding predictive factors in a consistent patient population. Further, excluding case reports, non-human studies, and those not meeting PRISMA or Cochrane standards enhance methodological robustness while limiting incomplete data on MRI or Ki-67 linked to pCR strengthens result validity.

Search Strategy and Study Selection

Two reviewers comprehensively searched for eligible studies from MEDLINE (PubMed), Cochrane Library, and MDPI from December 2010 till October 2024, focussing on cohort studies identifying the response of NAC in pCR utilizing Ki-67 and MRI. No language restriction was applied. Reference lists of all eligible trials were also searched to identify other studies. Duplicate studies were eliminated using EndNote 20.2.1 (Clarivate Analytics, Philadelphia, PA). A description of the keywords with Boolean operators used for each database is provided in Table 1.

**Table 1:** Search strategy and results obtained

Database	Search Strategy
PubMed (918 results)	("MRI" OR "Magnetic Resonance Imaging") AND ("tumor size" OR "tumour size" OR "residual tumor size") AND ("pathological complete response" OR "pCR") AND ("neoadjuvant chemotherapy")
	("Ki-67" OR "Ki67") AND ("pathological complete response" OR "pCR") AND ("neoadjuvant chemotherapy")
	("radiomics" OR "radiomic features" OR "radioclinicopathologic model") AND ("AUC" OR "Area Under the Curve") AND ("pathological complete response" OR "pCR") AND ("neoadjuvant chemotherapy")
	("MRI" OR "Magnetic Resonance Imaging") AND ("tumor size" OR "tumour size") AND ("Ki-67" OR "Ki67") AND ("radiomics" OR "radioclinicopathologic model") AND ("pathological complete response" OR "pCR") AND ("neoadjuvant chemotherapy")
	("predictors" OR "biomarkers") AND ("pathological complete response" OR "pCR") AND ("neoadjuvant chemotherapy") AND ("MRI" OR "Ki-67" OR "radiomics")
Cochrane Library (0 results)	("MRI" OR "Magnetic Resonance Imaging") AND ("tumor size" OR "tumour size") AND ("Ki-67" OR "Ki67") AND ("radiomics" OR "radioclinicopathologic model") AND ("pathological complete response" OR "pCR") AND ("neoadjuvant chemotherapy")
MDPI (0 results)	("MRI" OR "Magnetic Resonance Imaging") AND ("tumor size" OR "tumour size") AND ("Ki-67" OR "Ki67") AND ("radiomics" OR "radioclinicopathologic model") AND ("pathological complete response" OR "pCR") AND ("neoadjuvant chemotherapy")

Data Extraction

Two reviewers independently assessed the papers on Mendeley based on their titles and abstracts in accordance with the qualifying requirements. The full texts of the remaining papers were separately reviewed, and disagreements were addressed through conversation with a third reviewer. The following information was retrieved from the studies using a piloted Microsoft Excel sheet: the surname of the first author, publication year, design of the study, sample size (n), mean age with standard deviations, clinical presentations, and assessment criteria. The baseline characteristics offer an overview of the major components of the studies included in the meta-analysis (Table 2).

**Table 2:** Characteristics of the studies included in the meta-analysis TNBC-NST: Triple-Negative Breast Cancer - No Special Type; NAC: Neoadjuvant chemotherapy

Study	Year of Publication	Study Design	Sample Size	Mean Age	Clinical Presentation
Pesapane F et al. [13]	2021	Retrospective, single-center cohort	83	47.26±8.6	Breast Cancer receiving NAC
Kim YS et al. [14]	2021	Retrospective, single-center cohort	359	49±10	Stage II–III breast cancer
Harada TL et al. [15]	2019	Retrospective cohort	57	50±14.8 (pCR) and 51±11.9 (npCR)	TNBC-NST receiving NAC
Eom HJ et al. [16]	2017	Retrospective cohort	73	45±10.09	TNBC
Hottat NA et al. [17]	2023	prospective single-center study	47	53.4±14.75	invasive breast cancers
Yoen H et al. [18]	2023	Retrospective, single-center cohort	252	48.3 ± 10.7	TNBC who underwent NAC
Zhang Z et al. [19]	2014	Retrospective, single-center cohort	114	NA	Invasive breast cancer
Kim MJ et al. [20]	2014	Retrospective cohort study	35	48.49±10.04	Triple-negative breast cancer
Kim R et al. [21]	2019	Retrospective, randomized study	408	47.9 ± 6 9.6	Node-positive breast cancer treated with NAC following surgery
He M et al. [22]	2022	Prospective cohort	74	49.7±10.2	Stage II–III breast cancer

Methodological Quality Appraisal

National Institutes of Health quality assessment tool for observational and cross-sectional studies [25] consisting of 14 questions was used, each scored as Yes, No, or Not Applicable. The study quality was scored on a scale from zero to 14 and classified as poor, fair, or good based on the total score (zero-seven points as poor, 8-10 points as fair, or 11-14 points as good). For the NIH quality assessment tool, studies with mixed ratings across domains were assessed holistically. When a study had both "Yes" and "No" responses that created ambiguity in overall quality, an average rating approach was used. "Not Applicable" responses were excluded from the total score calculation to avoid penalizing studies for criteria irrelevant to their design, ensuring fairness in quality assessment. The NIH quality assessment tool was selected for evaluating observational cohort studies because it emphasizes assessing validity within cohort designs, focusing on factors such as population definition, exposure measurement, and outcome assessment. The table for quality assessment is attached in Appendix 1 of the study. For the observational cohort studies included, the Cochrane risk of bias in non-randomized studies - of interventions (ROBINS-I) tool [26] was employed to assess the risk of bias. The studies were evaluated across seven key domains: confounding, participant selection, intervention classification, deviations from intended interventions, missing data, outcome measurement, and selective reporting. ROBINS-I tool was used alongside the NIH tool to provide a more comprehensive bias assessment, particularly addressing confounding, participant selection, and outcome measurement, which are critical in observational studies lacking randomization. The results were represented through traffic light plots and summary bar charts using the Cochrane Risk-of-bias VISualization (Robvis) tool [27]. In the traffic light plot, green, yellow, and red indicators represented low, moderate, and high risk of bias, respectively, across domains. Expected findings included a low to moderate risk of bias in most domains, with occasional high bias in participant selection. Bar charts summarized these findings, highlighting areas where specific biases were prevalent across studies. To mitigate potential biases, we employed rigorous inclusion criteria, ensured consistency in data extraction, and performed sensitivity analyses wherever feasible.

Outcomes

The study's result focused on specific outcomes, particularly the association between the Ki-67 index, Ki positive count of individuals, tumor size measurement by MRI, and predictive radiomic-clinicopathological models in predicting pCR following NAC in breast cancer.

Statistical Analysis

The data were analyzed using Review Manager (RevMan) version 8.7.0, the Cochrane Collaboration (October 3, 2024) [26]. The random effects model was used to calculate mean differences (MD) and their corresponding 95% confidence intervals (95% CI) except for the analysis of radiomic-clinicopathologic models. The random-effects model was utilized due to the high estimated heterogeneity of the true effect sizes. The inverse variance (IV) method combined individual study results. For each synthesis, the I² index and the chi-square (Chi²) test were used for the assessment of heterogeneity, and a p-value of less than 0.05 was considered clinically significant for the included studies. Publication bias was assessed by the two-tailed Egger's regression intercept on the funnel plot where a p-value greater than 0.05 indicated no evidence of publication bias, which was performed for the studies showing predictive modeling for pCR. The random-effects model was employed to calculate the pooled risk ratio (RR) and corresponding 95% CI for dichotomous outcomes in analyzing radiomic-clinicopathologic models. This model was selected due to the substantial heterogeneity observed across the included studies, as indicated by the high I² value, reflecting significant variability in the effect sizes. The inverse variance (IV) method combined individual study results. Heterogeneity was assessed using both the I² statistic and the Chi² test, with a p-value of less than 0.05, indicating significant heterogeneity among the studies. A z-test for the overall effect was conducted, and a p-value of 0.05 was deemed statistically significant, demonstrating a difference in the risk between experimental and control groups. The risk ratios were visualized using a forest plot, where an RR of less than 1 indicated a reduced risk in the experimental group.

Results

Study Characteristics

**Figure 1:**
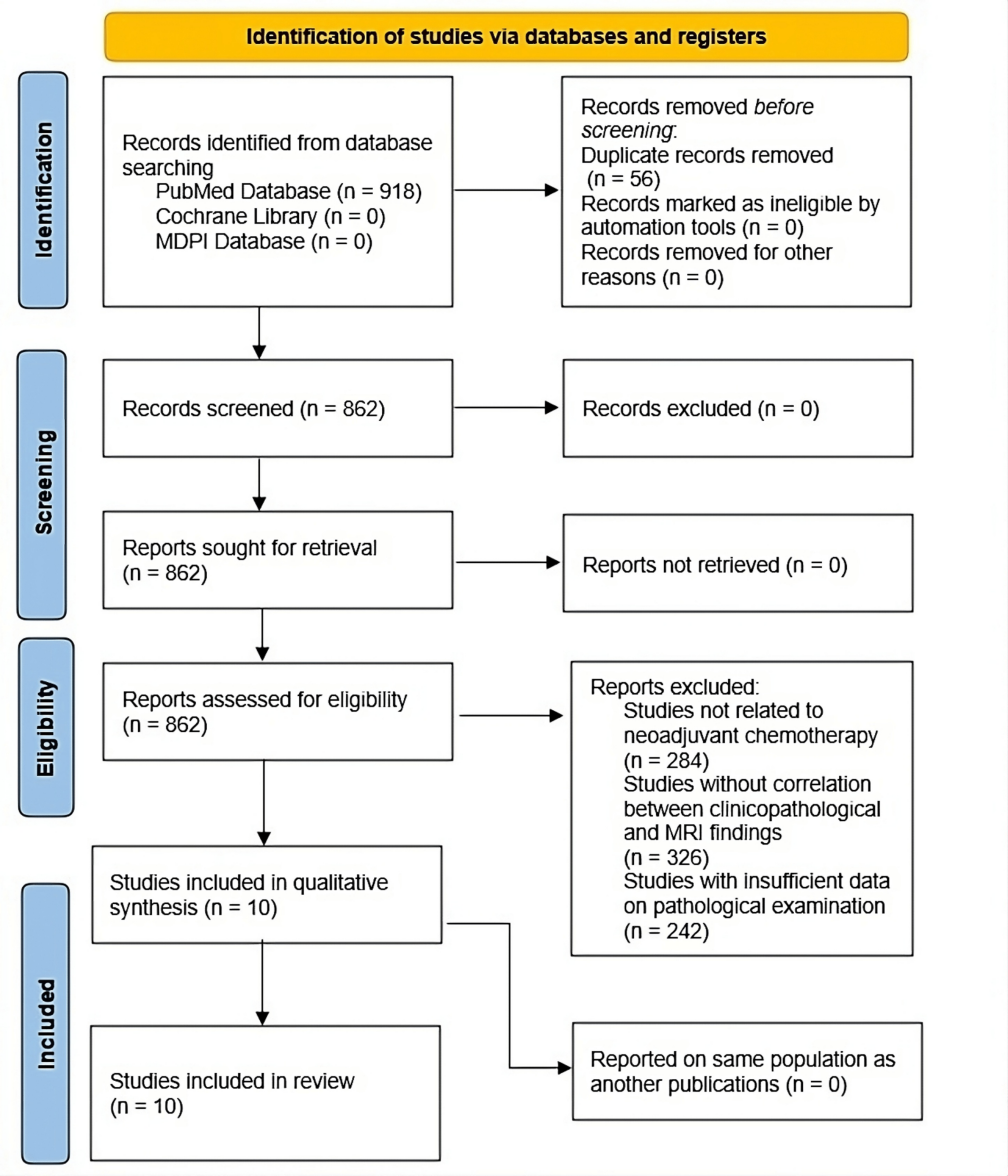
PRISMA flow chart PRISMA: Preferred Reporting Items for Systematic Reviews and Meta-Analysis

The PRISMA flow diagram in Figure 1 outlines the study selection process for the systematic review. The PRISMA flow diagram details the study selection process for researching the use of Ki-67 with MRI to predict pCR after NAC. Initially, 918 articles were identified from the PubMed database, while none were found in the Cochrane Library or MDPI databases. After removing 56 duplicate records, 862 unique records remained for screening, all of which were successfully retrieved. Upon assessing eligibility, 284 reports were excluded for being unrelated to NAC, 326 lacked correlations between clinicopathological and MRI findings, and 242 had unclear methodology or insufficient data. Ultimately, 10 studies met the inclusion criteria and were included in the qualitative synthesis and final review. This comprehensive process highlights the rigorous methodology employed to identify relevant studies examining the relationship between Ki-67, MRI, and pCR following NAC.

Risk of Bias

**Figure 2:**
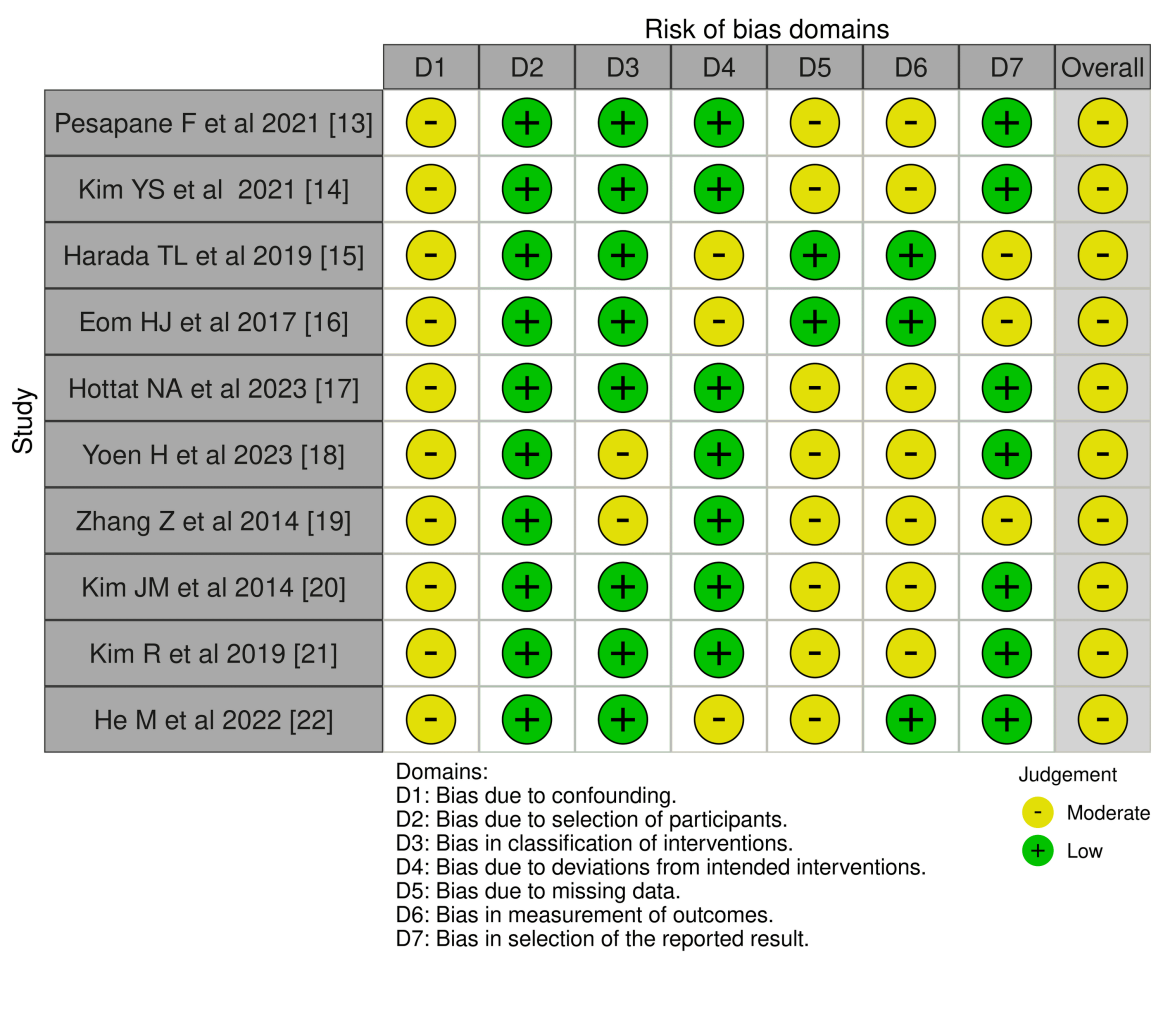
Risk of bias traffic light plot for the included studies

The traffic plot in Figure 2 provides an overview of the risk of bias across several studies evaluating the predictive role of Ki-67 and MRI in determining complete pCR following NAC. Studies such as Pesapane et al. (2021) [13], Harada et al. (2019) [15], and Eom et al. (2017) [16] generally followed standardized protocols for MRI assessments, often using independent or blinded radiologists, ensuring consistency and reducing measurement bias. Participant selection was appropriate across most studies, with clearly defined inclusion criteria based on NAC treatment and imaging results. However, confounding remains a concern, as not all studies adequately controlled for variability in chemotherapy regimens, patient characteristics, or tumor biology. Handling of missing data was inconsistent, with some participants lacking MRI follow-up or Ki-67 readings, affecting outcome reliability. While most studies maintained intervention consistency, minor deviations occurred in a few, particularly in chemotherapy protocols. Additionally, selective reporting of significant results was noted in several studies, which may introduce bias. Overall, while the studies provide useful insights, moderate limitations exist, especially in areas like confounding, missing data, and selective reporting, suggesting that their conclusions should be interpreted with caution.

**Figure 3:**
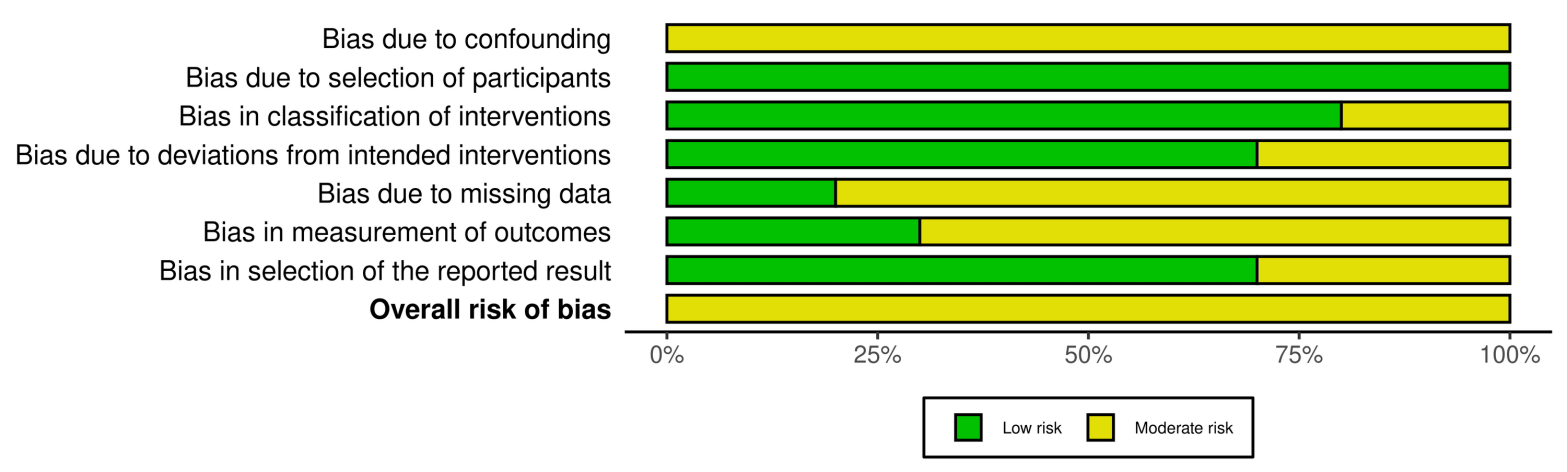
Risk of bias overall summary of the included studies

The summary graph in Figure 3 highlights the distribution of risk across key domains for multiple studies assessing the predictive value of Ki-67 and MRI for complete pCR after NAC. Confounding is a prevalent issue, with most studies exhibiting moderate risk due to limited control over factors like chemotherapy protocols and patient variability. Participant selection shows lower risk, suggesting appropriate inclusion criteria across studies. Classification of interventions is well-handled, with only minor inconsistencies. Deviations from intended interventions, such as variations in chemotherapy regimens, pose some risks but remain largely controlled. Missing data presents a moderate risk, reflecting incomplete follow-ups or unavailable Ki-67 results in several cases. Measurement of outcomes has lower risk, indicating that imaging protocols were standardized and often conducted by blinded radiologists. However, many studies are prone to selective reporting, focusing on significant results, which contributes to the moderate overall risk of bias. This emphasizes the need for a cautious interpretation of the findings.

Ki-67 Index Percentage

**Figure 4:**
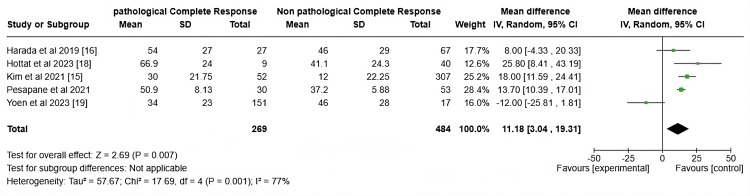
Forrest plots of studies showing Ki-67 Index percentages

This forest plot in Figure 4 illustrates the comparison of Ki-67 levels between patients who achieved pCR and those with non-pCR across five different studies. The mean difference represents the difference in the Ki-67 index among the pCR and non-pCR groups. A negative mean difference implies that patients with pCR had lower Ki-67 percentages compared to those without pCR. The studies by Harada et al. (2019) [15], Kim et al. (2021) [14], and Pesapane et al. (2021) [13] show positive mean differences, indicating higher Ki-67 levels in patients who achieved pCR. The study by Yoen et al. (2023) [18] shows a negative mean difference, which suggests lower Ki-67 levels in the pCR group compared to the non-pCR group. The study by Hottat NA et al. (2023) [17] has the largest mean difference (25.80), indicating a significant association between higher Ki-67 and pCR. The overall pooled mean difference is 11.18 (95% CI: 3.04, 19.31), which is statistically significant (z = 2.69, P = 0.007). This suggests that, on average, patients who achieve pCR have higher Ki-67 levels compared to those who do not. The substantial heterogeneity (I² = 77%) indicates considerable variability among the studies, possibly due to differences in patient populations, treatment protocols, or the definition of Ki-67 cut-offs. The plot suggests that high Ki-67 is associated with a better response towards pCR, with most studies reporting that patients who achieve pCR tend to have higher pretherapeutic Ki-67 levels. This reinforces the idea that Ki-67 could serve as a valuable predictive marker for response to NAC in breast cancer patients. The findings suggest that higher Ki-67 levels may predict a favorable response to NAC in breast cancer patients, as evidenced by the association between elevated Ki-67 and pCR. Clinically, this implies that assessing Ki-67 levels before NAC could help oncologists identify patients more likely to benefit from such treatment, potentially guiding personalized therapy. Moreover, the substantial heterogeneity among studies emphasizes the need for standardizing Ki-67 assessment methods.

Ki-67 Positive Count

**Figure 5:**
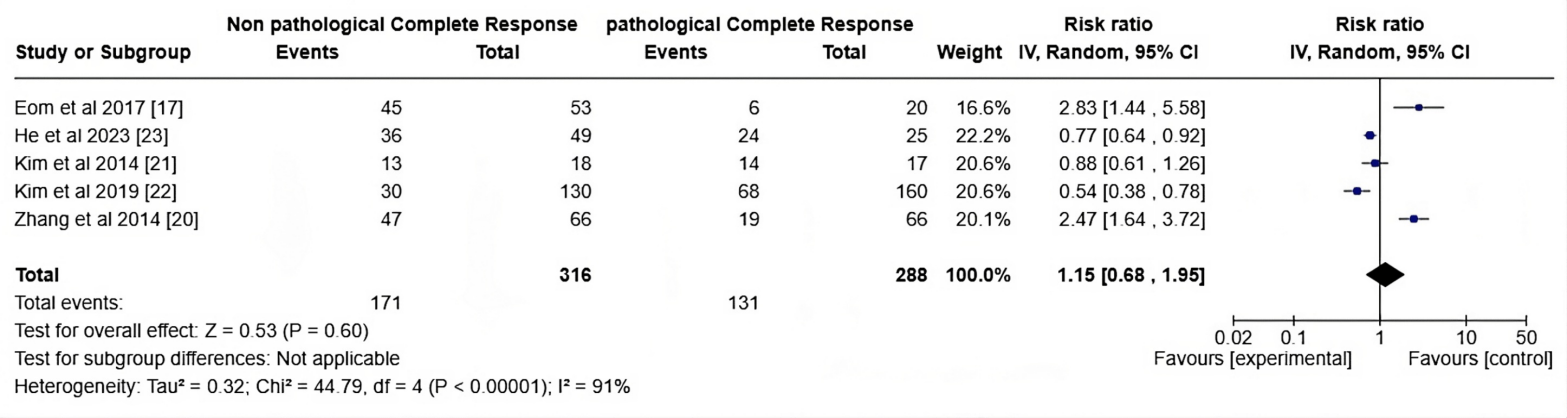
Forrest plot on the positive Ki-67 count of individuals post-NAC NAC: Neoadjuvant chemotherapy

The meta-analysis presented in the forest plot Figure 5 evaluates the risk ratio between non-pCR and pCR across five studies. The total number of events in the non-pCR group is 171, compared to 131 in the pCR group. The pooled risk ratio, calculated using a random-effects model, is 1.15 (95% CI: 0.68, 1.95), suggesting no significant association between the two groups (p = 0.60). Significant heterogeneity is evident (I² = 91%, p<0.01), implying variability across the studies. Individual study risk ratios vary substantially, with Eom et al. (2017) [16] showing the highest risk (RR: 2.83, 95% CI: 1.44, 5.58), while Kim et al. (2019) [21] and Zhang et al. (2014) [19] also show substantial contributions. This shows that the individuals with positive Ki-67 count may be a predictor for pCR, though it is not statistically significant. Clinically, Ki-67 may be useful as part of a multi-biomarker approach to guide personalized NAC decisions, especially if standardization of Ki-67 measurement is achieved.

MRI Tumor Size

**Figure 6:**
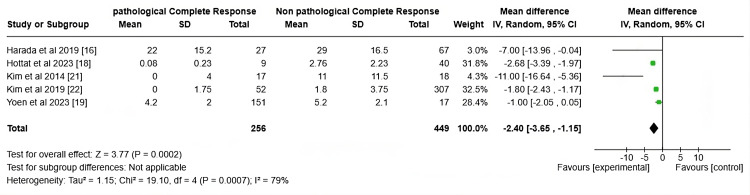
Forrest plot on the tumor size measured by MRI

The forest plot in Figure 6 presents the mean difference (MD) between pCR and non-pCR groups across five studies focusing on tumor size post-NAC. The overall mean difference is -2.40 (95% CI: -3.65 to -1.15), favoring the pCR achievement, indicating that those achieving pCR had a statistically significant reduction in the tumor size compared to the non-pCR group (z=3.77, p=0.0002). However, notable heterogeneity (I² = 79%) suggests considerable variability between the studies. Despite this, the results consistently favor the experimental intervention, suggesting that it may contribute to improved outcomes, possibly related to tumor shrinkage or Ki-67 reduction. Clinically, this supports using tumor size reduction and possibly Ki-67 levels as biomarkers to assess NAC efficacy and predict pCR. Monitoring these metrics could guide treatment adjustments, optimizing outcomes for patients who are responsive to chemotherapy.

MRI-Rim Enlargement

**Figure 7:**
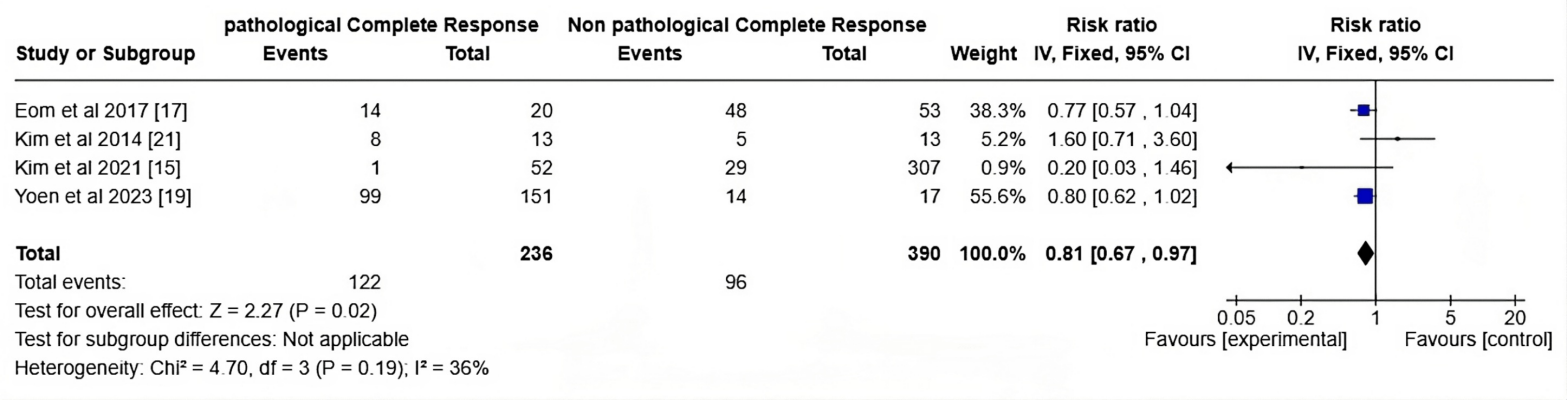
Forest plot on risk ratio involved with tumor rim enlargement post-NAC NAC: Neoadjuvant chemotherapy

The forest plot in Figure 7 displays the risk ratio (RR) for MRI rim enlargement counts between individuals who achieved pCR and those who did not (non-pCR). The overall pooled RR is 0.81 (95% CI: 0.67, 0.97), with a statistically significant p-value (p = 0.02). This indicates that individuals in the experimental group (those who achieved pCR) had a 19% lower risk of having high MRI rim enlargement counts compared to those in the control group (non-pCR). For the study by Eom et al. (2017) [16], the RR was found to be 0.77 (95% CI: 0.57, 1.04), suggesting a slight but non-significant association between reduced MRI rim enlargement and pCR. Kim et al. (2014) [20] showed an RR value of 1.60 (95% CI: 0.71, 3.60), indicating no clear association, as the CI crosses 1. Kim et al. (2021) [14] demonstrated an RR of 0.20 (95% CI: 0.03, 1.46), indicating a lower likelihood of MRI rim enlargement in the pCR group, but it is not statistically significant. The RR of Yoen et al. [18] was 0.80 (95% CI: 0.62, 1.02), again suggesting a lower risk of MRI rim enlargement in the pCR group, though the result is not statistically significant. The I² statistic, being 36%, indicates moderate heterogeneity across the studies, meaning that while there is some variability in the study results, it is not excessive, and the pooled result is reasonably reliable. Clinically, MRI findings can help tailor therapy, guiding clinicians in optimizing treatment plans. However, integrating MRI with other diagnostic markers is crucial for comprehensive assessments and enhancing predictive accuracy in patient management.

Radiomics-Clinicopathological Models

**Figure 8:**
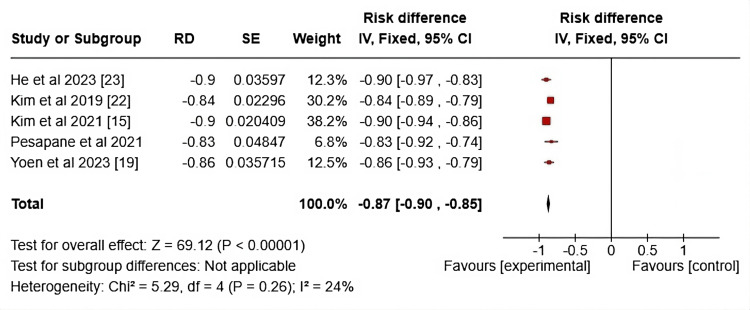
Forrest plots on the various AUCs of the radiomic-clinicopathological models RD: Risk difference; AUC: Area under the receiver operating characteristic curve

This forest plot in Figure 8 shows the risk difference (RD) between experimental and control groups across five studies, analyzing their association with an outcome (Ki-67 and pCR). Each study's AUC is plotted as a risk difference, and a 95% CI is plotted. All studies report a negative risk difference, meaning that the experimental group had a higher likelihood of achieving the desired outcome (likely pCR) compared to the control group. The overall risk difference across studies is -0.87 (95% CI: -0.90 to -0.85), indicating a statistically significant reduction in risk, favoring the experimental intervention. The test for heterogeneity (I² = 24%) suggests low-to-moderate variability between studies, meaning the results are generally consistent across the studies included. The test for overall effect (Z = 69.12, P < 0.00001) shows a strong statistical significance, reinforcing the conclusion that the experimental intervention substantially reduces the risk compared to the control. Clinically, these results highlight the potential for adopting these experimental approaches to optimize pCR rates, potentially guiding more effective treatment strategies and better prognostic outcomes for patients undergoing neoadjuvant therapy.

**Figure 9:**
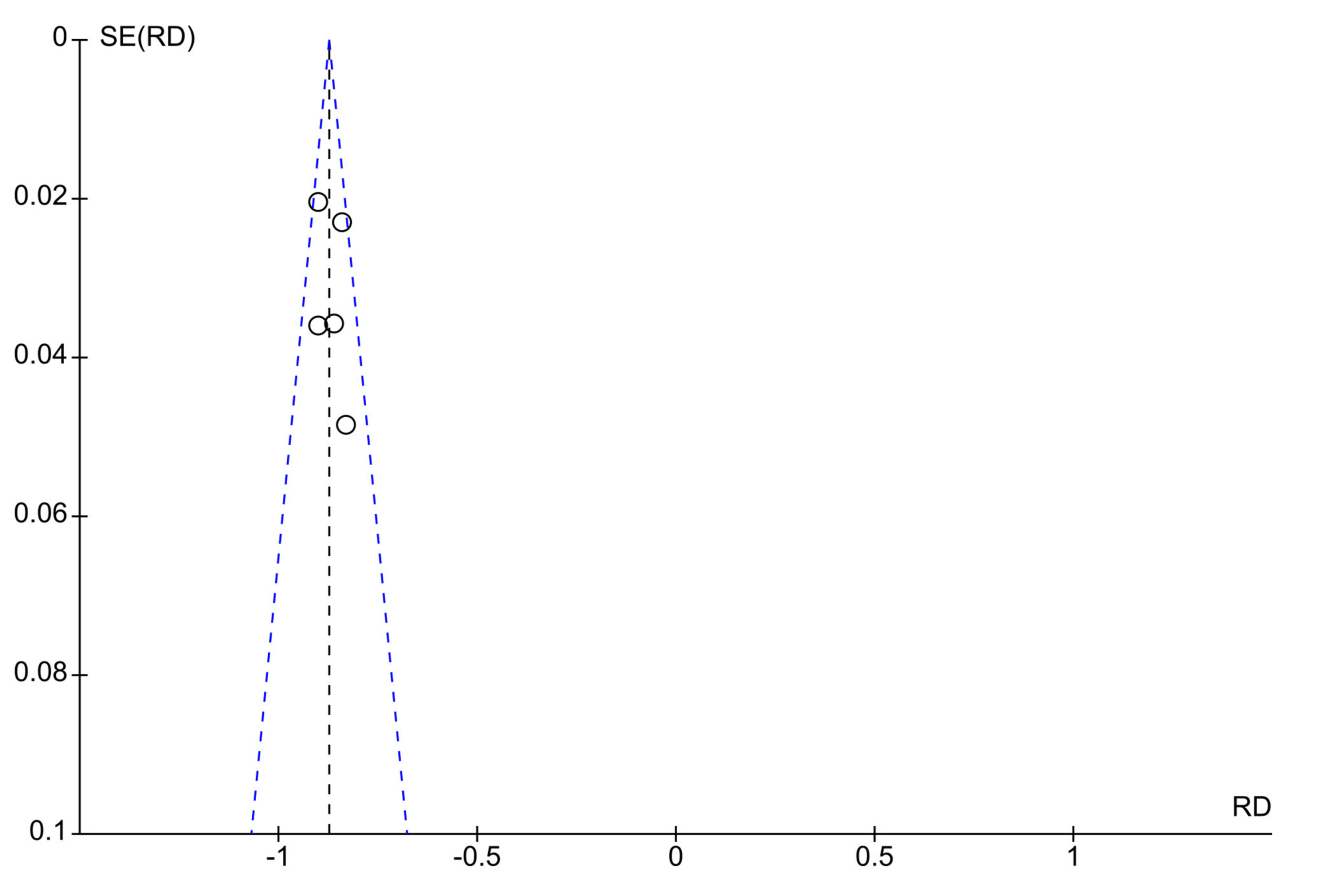
Funnel plots on the studies showing radiomic-clinicopathological models

The funnel plot displayed in Figure 9 evaluates the publication bias in studies using MRI and Ki-67 to predict pCR in patients undergoing NAC, which utilized the radiomic-pathological models. The x-axis represents the risk difference, while the y-axis shows the standard error of the risk difference. The plot appears symmetrical, with most studies, including Kim et al. (2021) [14], Kim et al. (2019) [21], and Pesapane et al. (2021) [13], clustered close to the funnel's peak, indicating smaller SE values and lower variance in effect sizes. These studies are situated near the centerline (RD = 0), suggesting a balanced distribution of results around the mean. There is no significant asymmetry, which suggests minimal publication bias. However, there is slight dispersion in the lower SE region, particularly for the Kim et al. (2021) [14] and Pesapane et al. (2021) [13] studies, indicating potential variability in predictive accuracy for pCR across these studies.

Discussion

Achieving pCR following NAC is linked with better event-free survival and overall survival, particularly for triple-negative and HER2-positive breast cancer [28]. A minimal Invasive biopsy can diagnose a pCR accurately, given a histopathological sample [29]. An efficient approach to identify pCR post-NAC is through the Ki-67 index and MRI. Thus, for our analysis, we included the pooled data of studies showing the efficacy of MRI and biomarker Ki-67 for precise identification of pCR.

Ki-67 and pCR

The comparison of our findings with Chen et al. [30] and Tao et al. [31] suggests that Ki-67 remains a valuable marker for predicting response to NAC, though the predictive direction (higher vs. lower Ki-67 levels) may vary depending on specific study designs and populations. Both our study and Chen et al.'s [30] meta-analysis affirm that patients with higher pre-treatment Ki-67 levels tend to achieve pCR more frequently, especially in the context of treatments involving anthracyclines and taxanes [30]. The substantial heterogeneity in our study (I² = 77%) and other studies points to the necessity of refining Ki-67 cut-off values, standardizing the measurement methods, and considering other tumor characteristics (such as hormone receptor and HER2 status) to further improve its utility as a predictive marker. Despite this variability, the consistency of Ki-67's predictive capacity across different study designs and populations strengthens the argument for incorporating Ki-67 into clinical decision-making for selecting patients most likely to benefit from NAC.

MRI and pCR

The findings from Chang et al. [32], Teifke et al. [33], and our study align in highlighting MRI-measured rim enlargement as a marker of aggressive tumor behavior and poorer clinical outcomes. Chang et al. [32] and Teifke et al. [33] both mention the importance of early dynamic enhancement peaks and washout rates, with these features being linked to aggressive tumor characteristics. Though our study focused specifically on rim enlargement rather than enhancement kinetics, the correlation between rim enlargement and non-pCR implies similar underlying dynamic characteristics, such as fast early enlargement and washout, which are typical of aggressive malignancies. In particular, the association of rim enhancement with factors such as high tumor grade, negative hormone receptor status, lymph node metastasis, and increased micro-vessel density is supported by our meta-analysis's inverse relationship between MRI rim enlargement and pCR. This suggests that rim enhancement may not only be indicative of poor prognostic features but also a predictor of reduced treatment response, providing valuable insights for clinical decision-making in breast cancer management.

The forest plot in Figure 6 indicates a significant mean difference of -2.40 (95% CI: -3.65 to -1.15) between the pCR and non-pCR groups concerning tumor size post-NAC, highlighting tumor shrinkage as a potential predictor of pCR. Patients achieving pCR displayed a substantial reduction in tumor size compared to non-pCR individuals (Z = 3.77, P = 0.0002). This aligns with the understanding that tumor size is a critical predictor of therapeutic response, where a more significant reduction correlates with a better overall prognosis. Tumor size reduction has long been considered a surrogate marker for the effectiveness of NAC, particularly in breast cancer. Achieving pCR often signals a favorable prognosis, including lower recurrence rates and improved survival outcomes. However, the notable heterogeneity (I² = 79%) reflects differences between studies, likely due to variations in tumor biology, treatment regimens, and patient populations. Despite this variability, tumor size remains a reliable predictor of pCR and could guide therapeutic decision-making in clinical practice.

Radiomic-clinicopathologic Combination for pCR 

Studies demonstrate that predictive models incorporating Ki-67 and MRI metrics have high validity in forecasting pCR in breast cancer patients undergoing NAC. Models like those by He et al. [22] and Kim et al. (2021) [14] show robust predictive accuracy, with AUC values nearing 0.90 when using MRI-based metrics alongside Ki-67, particularly after initial NAC cycles. These findings are reinforced by Pesapane et al. [13], who showed that combining radiomic MRI features with clinical data, including Ki-67, improves predictive capacity, suggesting that MRI alone is moderately predictive but yields a more comprehensive model when coupled with biological markers. Including Ki-67, a marker of cell proliferation, provides essential pathological insight into tumor aggressiveness, while MRI captures dynamic changes in tumor morphology and vascularity. Together, they form an integrated approach that captures both molecular and structural tumor profiles, enabling highly accurate predictions. Across studies, the predictive validity of these models supports their use in clinical decision-making, allowing for tailored treatment strategies that enhance response outcomes and avoid unnecessary treatments. This integrated modeling approach is a promising tool for personalized breast cancer care.

Limitations

Although this study pro­vides some interesting insights into the processes of meaning-making towards combining pathological and radiological examinations for NAC, it has several limitations. First, differences in the methodology of imaging analysis and Ki-67 index assessment for primary and metastatic lesions between the studies may represent a source of variability. The considerable degree of inconsistency (I² > 75%) indicates that variations in the NAC regimens or chemotherapy dosing, tumor types, or patients' characteristics in studies may affect prediction accuracy. Furthermore, while MRI and Ki-67 are informative, they do not provide a complete picture of the molecular state of breast cancer and may miss other consequential biomarkers. The use of such data sources is relevant for some models and also raises issues of selection and information bias, thereby limiting the external validity of such conclusions to broader clinical practice settings. Moreover, despite the fact that Ki-67 is frequently connected with pCR, its value as the only criterion is questionable, and its variations throughout NAC are not well understood. Standardization of imaging and biomarker assessment procedures would be relevant to improve feasibility and generalizability in future research.

Future Prospects

Molecular-morphological scoring based on preoperative MRI and Ki-67 would require prospective multi-center studies to confirm the value of our model in predicting pCR within different populations and clinical settings. Improvement in aspects like forming cross-sectional imaging measurement criteria and cross-sectional MRI sequence standardization, besides the cross-sectional value of the Ki-67 scoring system, will be important characteristics for reproducibility to be enhanced. Generalizing the models with other sets of biomarkers, that is, genomic and/or transcriptomic, could potentially provide better estimates of tumor response. The utility of dynamic changes in Ki-67 and MRI features may be investigated across other NAC stages to derive additional information regarding treatment outcomes. Furthermore, there is a potential for enhancement of the prediction model by using MRI radiomic features along with clinicopathologic characteristics supported by artificial intelligence. With these innovations, it is possible to take such tailored therapeutic approaches to the next level to make them a part of standard processes of breast cancer treatment for women.

## Conclusions

This study underscores the transformative potential of combining MRI and Ki-67 biomarker levels to predict pCR in breast cancer patients undergoing NAC. Key findings reveal that higher Ki-67 levels, alongside MRI indicators such as tumor size and rim enhancement, are robust predictors of pCR, achieving high predictive accuracy across multiple studies. This integrated approach provides a more comprehensive view of tumor biology and treatment response, laying a foundation for more targeted and personalized therapeutic strategies. Future studies should focus on refining these models by incorporating additional biomarkers and AI-driven radiomic analysis, standardizing imaging protocols, and validating findings in diverse populations through prospective trials. This approach could ultimately streamline decision-making in clinical oncology, minimizing overtreatment and improving outcomes for high-risk breast cancer patients, thereby marking a significant advancement in personalized cancer care.

## Appendices

**Table 3:** Quality Assessment of the included studies

Criteria	Eom et al. (2017) [16]	Harada et al. (2020) [15]	He et al. (2023) [22]	Hottat et al. [17]	Kim et al. (2019) [21]	Kim et al. (2014) [20]	Kim et al. (2021) [14]	Pesapane et al. (2021) [13]	Yoen et al. (2023) [18]	Zhang et al [19]
1. Research question/objective clearly stated?	Yes	Yes	Yes	Yes	Yes	Yes	Yes	Yes	Yes	Yes
2. Study population specified and defined?	Yes	Yes	Yes	Yes	Yes	Yes	Yes	Yes	Yes	Yes
3. Participation rate ≥ 50%?	Not available	Not available	Yes	Yes	Yes	Yes	Not Available	Not Available	Not Available	Not Available
4. Uniform population and criteria applied?	Yes	Yes	Yes	Yes	Yes	Yes	Yes	Yes	Yes	Yes
5. Sample size justification provided?	No	No	Yes	No	No	No	No	Yes	No	No
6. Exposure measured before outcome?	Yes	Yes	Yes	Yes	Yes	Yes	Yes	Yes	Yes	Yes
7. Timeframe sufficient for exposure-outcome association?	Yes	Yes	Yes	Yes	Yes	Yes	Yes	Yes	Yes	Yes
8. Different exposure levels examined?	Yes	Yes	Yes	Yes	Yes	Yes	Yes	Yes	Yes	Yes
9. Exposure measures defined, valid, and reliable?	Yes	Yes	Yes	Yes	Yes	Yes	Yes	Yes	Yes	Yes
10. Exposure assessed more than once?	No	No	Yes	Yes	Yes	Yes	Not Available	No	Not Available	Yes
11. Outcome measures defined, valid, and reliable?	Yes	Yes	Yes	Yes	Yes	Yes	Yes	Yes	Yes	Yes
12. Outcome assessors blinded?	Not available	Not available	Yes	Not Available	Not Available	Not Available	Not Available	Not Available	Not Available	Yes
13. Loss to follow-up ≤ 20%?	Yes	Not available	Yes	Yes	Yes	Yes	Not Available	Not Available	Not Available	Not Available
14. Confounding variables adjusted for?	Yes	No	Yes	Yes	Yes	Yes	Yes	Yes	Yes	Yes
Total Score	10.00	9.00	14.00	12	12	12	10	11	10	11
Quality Classification	Fair	Fair	Good	Good	Good	Good	Fair	Good	Fair	Good
